# Rapid relapse in triple-negative breast cancer: clinical patterns, platinum resistance, and implications for clonal evolution

**DOI:** 10.3389/fphar.2026.1889398

**Published:** 2026-07-10

**Authors:** Tao Ma, Shuang-Long Cai, Hong-Dan Chen, Jin Zhang

**Affiliations:** 1 The Third Department of Breast Cancer, Tianjin Medical University Cancer Institute & Hospital, National Clinical Research Center for Cancer, Tianjin, China; 2 Tianjin’s Clinical Research Center for Cancer, Tianjin Medical University Cancer Institute & Hospital, Tianjin, China; 3 Key Laboratory of Breast Cancer Prevention and Therapy, Tianjin Medical University, Ministry of Education, Tianjin, China; 4 Key Laboratory of Cancer Prevention and Therapy, Tianjin Medical University Cancer Institute & Hospital, Tianjin, China; 5 Key Laboratory of Cancer Immunology and Biotherapy, Tianjin, China; 6 Department of Thyroid and Breast Surgery, Comprehensive Breast Health Center, The Lishui Hospital of Wenzhou Medical University, The First Affiliated Hospital of Lishui University, Lishui People’s Hospital, Lishui, Zhejiang, China; 7 First Department of Cadre Clinic, Fuzhou University Affiliated Provincial Hospital, Fujian Provincial Hospital, Fuzhou, Fujian, China

**Keywords:** clonal evolution, prognosis, rapid relapse, triple-negative breast cancer, tumor heterogeneity

## Abstract

**Background:**

Triple-negative breast cancer (TNBC) is characterized by high heterogeneity and poor prognosis. A particularly aggressive subset, termed ‘rapid relapse’ TNBC (rrTNBC), is defined by distant metastasis or death within 24 months of diagnosis. Understanding the unique recurrence patterns and prognostic determinants of rrTNBC is crucial for deciphering tumor evolution and optimizing therapeutic strategies. This study aims to delineate the clinicopathological features, recurrence patterns, and prognostic outcomes of rrTNBC compared to slow relapse TNBC (srTNBC).

**Methods:**

We retrospectively analyzed 638 postoperative patients with recurrent or metastatic TNBC treated at Tianjin Medical University Cancer Institute & Hospital. Patients were categorized into rrTNBC (relapse ≤24 months, n = 478) and srTNBC (relapse >24 months, n = 160). Clinicopathological variables, recurrence sites, and survival outcomes, including disease-free survival (DFS), progression-free interval (PFI) after first salvage therapy, post-recurrence survival (PRS), and overall survival (OS), were compared between the two groups.

**Results:**

Compared to srTNBC, rrTNBC was associated with higher TNM stage, T stage, N stage, and a lower proportion of stromal tumor-infiltrating lymphocyte expression (all p < 0.001). Regarding recurrence patterns, rrTNBC patients were more likely to present with visceral (74.90% vs. 22.50%, p < 0.001) and brain metastases (10.88% vs. 0.62%, p < 0.001) as the first event, whereas srTNBC patients had a higher incidence of first relapse in the chest wall or regional lymph nodes (90.00% vs. 62.13%, p < 0.001). Consequently, rrTNBC was characterized by a significantly higher rate of direct distant metastasis at first recurrence (93.10% vs. 28.12%, p < 0.001). Prognostically, rrTNBC patients had markedly worse outcomes across all metrics compared to srTNBC patients, including median disease-free survival (13.4 vs. 26.6 months), median PFI after first-line salvage therapy (2.27 vs. 8.43 months), median PRS (12.1 vs. 23.5 months), and median overall survival (26.5 vs. 52.0 months) (all p < 0.001). In the platinum-based first-line salvage therapy subgroup (n = 369), rrTNBC patients (n = 267) consistently demonstrated significantly shorter DFS, PFI, PRS, and OS compared to srTNBC patients (n = 102) (all p < 0.001).

**Conclusion:**

rrTNBC represents a distinct, highly aggressive phenotype with a predilection for visceral and brain metastasis and a dismal response to conventional platinum-based salvage therapy. These findings are consistent with the aggressive clonal evolution within rrTNBC and highlight an urgent need for novel therapeutic strategies to be prospectively evaluated in this high-risk population.

## 1 Introduction

Triple-negative breast cancer (TNBC), accounting for approximately 10%–15% of all breast cancer cases, is responsible for a disproportionately high number of breast cancer-related deaths due to its aggressive biological behavior and lack of effective therapeutic targets (Bray et al., 2024; Foulkes et al., 2010). Unlike hormone receptor-positive subtypes, TNBC is characterized by higher histological grade, increased proliferative index, and a pronounced propensity for early recurrence, with the majority of relapses occurring within the first 3 years following diagnosis (Dent et al., 2007; Bauer et al., 2007). The metastatic pattern of TNBC also differs significantly, showing a predilection for visceral organs such as the lung and liver, as well as the central nervous system, while bone metastases are relatively less common (Lin et al., 2012; Dent et al., 2009). This aggressive clinical trajectory is largely attributed to the high intratumoral heterogeneity and genomic instability that drive rapid clonal expansion and therapeutic resistance (Aguilar-Mahecha et al., 2023; McGranahan and Swanton, 2017). Despite advances in neoadjuvant and adjuvant chemotherapy, a substantial subset of TNBC patients experiences disease progression within 24 months of initial diagnosis, a phenomenon that has been increasingly recognized as a distinct clinical entity warranting dedicated investigation (Liedtke et al., 2008; Yin et al., 2009).

Within the heterogeneous landscape of TNBC, the concept of “rapid relapse” has emerged as a critical prognostic indicator. Recent studies have attempted to characterize this aggressive subset. For instance, Asad and colleagues identified sociodemographic factors, including younger age (<50 years), Medicaid insurance, and lower income, as independent predictors of rapid relapse in a large multi-institutional cohort (Asad et al., 2021). Subsequent genomic analyses by Zhang et al. revealed that rapid relapse TNBC (rrTNBC) is associated with significantly lower expression of immune-related gene signatures, suggesting an immunosuppressive tumor microenvironment (TME) that may permit unchecked clonal proliferation (Zhang et al., 2021). Furthermore, Obeng-Gyasi et al. demonstrated that lack of surgical intervention and certain socioeconomic disparities are linked to higher rates of rapid relapse (Obeng-Gyasi et al., 2021). However, these existing studies have primarily focused on baseline sociodemographic and limited genomic features, leaving a substantial gap in our understanding of the detailed recurrence patterns, metastatic organotropism, and-most critically-the response to conventional salvage therapies in rrTNBC patients. The clinical implications of these gaps are profound, as they hinder the development of risk-stratification models and effective treatment strategies for this high-risk population (Harbeck and Gnant, 2017).

The present study was therefore designed to address these critical knowledge gaps by providing a comprehensive, real-world characterization of rrTNBC. Leveraging a large, single-institutional cohort of 638 recurrent/metastatic TNBC patients, we aimed to: (i) compare the clinicopathological features, including sTIL expression and TNM staging, between rrTNBC and slow relapse TNBC (srTNBC); (ii) delineate the distinct patterns of first recurrence or metastasis, with a particular focus on visceral, brain, and locoregional relapse; and (iii) evaluate the prognostic outcomes, including overall survival (OS) and post-recurrence survival (PRS), with a specific subgroup analysis of patients receiving first-line platinum-based salvage therapy after failure of anthracycline/taxane-based regimens. By elucidating the unique clinical trajectory and treatment resistance profile of rrTNBC, this study seeks to provide a framework for understanding the underlying clonal evolution driving this aggressive phenotype and to inform the rational design of novel therapeutic interventions, including antibody-drug conjugates (ADCs) and immunotherapy combinations, for patients with this devastating disease (Schmid et al., 2018; Bardia et al., 2021).

## 2 Methods

### 2.1 Study population

We screened all patients with a pathological diagnosis of TNBC who received treatment at Tianjin Medical University Cancer Institute and Hospital between 1 January 2016, and 31 December 2021. Eligibility criteria for this analysis included: (i) female patients aged 18 years or older; (ii) histologically confirmed invasive breast carcinoma with negative expression of estrogen receptor (ER), progesterone receptor (PR), and human epidermal growth factor receptor 2 (HER2) according to American Society of Clinical Oncology/College of American Pathologists guidelines (Wolff et al., 2018); (iii) development of disease recurrence or metastasis after initial curative-intent treatment (surgery with or without (neo)adjuvant chemotherapy); and (iv) complete clinical, pathological, and follow-up data available. ER and PR negativity was defined as <1% positive tumor cell nuclei by immunohistochemistry (IHC). HER2 negativity was defined as IHC 0 or 1+, or IHC 2+ with negative fluorescence *in situ* hybridization (FISH). Patients with stage IV disease at initial diagnosis, bilateral breast cancer, other synchronous malignancies, or incomplete medical records were excluded.

### 2.2 Definitions and group assignment

The primary exposure variable was the time from initial diagnosis to first documented disease recurrence or metastasis. Consistent with prior literature (Asad et al., 2021; Zhang et al., 2021), we defined rapid relapse TNBC (rrTNBC) as distant metastasis or death occurring within 24 months (≤24 months) of the initial breast cancer diagnosis. Slow relapse TNBC (srTNBC) was defined as distant metastasis or death occurring after 24 months (>24 months) from initial diagnosis. This cut-off was chosen based on previous reports indicating that the peak risk of recurrence in TNBC occurs within the first 2–3 years (Dent et al., 2007; Bauer et al., 2007).

### 2.3 Data collection and clinicopathological variables

Demographic and clinicopathological data were extracted from electronic medical records by trained research staff blinded to the study hypothesis. The following variables were collected: age at diagnosis (<50 vs. ≥50 years), menopausal status, family history of breast/ovarian cancer, surgical approach (mastectomy vs. breast-conserving surgery), pathological pattern, postoperative pathological TNM staging (I, II, or III based on the 8th edition of the AJCC Cancer Staging Manual), postoperative pathological T staging (T0/Tis + T1, T2, T3+T4), postoperative pathological N staging (N0, N1, N2, N3), tumor grade (G1+G2 vs. G3), lymph-vascular invasion, stromal tumor-infiltrating lymphocyte (sTIL) expression levels, HER2 expression level (IHC 0, 1+, or 2+/FISH-), Ki-67 proliferation index (≤20% vs. >20%), p53 expression status, CK5/6 expression, EGFR expression, and receipt of radiotherapy.

Stromal TILs were assessed on hematoxylin and eosin-stained sections of surgical resection specimens according to the international TILs Working Group guidelines (Salgado et al., 2015). sTIL expression was categorized into three groups: low (≤10%), intermediate (10% < sTIL ≤40%), and high (>40%), as previously described.

### 2.4 Recurrence and metastasis characteristics

The primary outcomes of interest for the descriptive analysis were the patterns of first recurrence or metastasis event. These were classified as: (i) visceral metastasis (liver or lung), (ii) bone metastasis, (iii) brain metastasis, (iv) chest wall or regional lymph nodes relapse (including ipsilateral breast, chest wall, axillary, internal mammary, or supraclavicular lymph nodes), and (v) the patterns of first recurrence or metastasis, categorized as either “direct distant metastasis” (defined as distant metastasis as the first recurrence or metastasis event), with or without concurrent chest wall or regional lymph nodes recurrence) or “chest wall or regional lymph node recurrence prior to distant metastasis”. Distant metastasis including visceral metastasis, bone metastasis, brain metastasis.

### 2.5 Survival outcomes

The primary prognostic outcomes were disease-free survival (DFS), progression-free interval (PFI) after first-line salvage therapy, post-recurrence survival (PRS), and overall survival (OS). **DFS** was defined as the time from the date of definitive surgery to the date of first documented recurrence or metastasis, or death from any cause, whichever occurred first (Hudis et al., 2007). **PFI** was defined as the time from the initiation of first-line salvage therapy for recurrent/metastatic disease to the date of disease progression (radiographic or clinical) or death. **PRS** was calculated as the time from the date of first recurrence/metastasis to the date of death from any cause. **OS** was defined as the time from the date of initial breast cancer diagnosis to the date of death from any cause or last follow-up (Gourgou-Bourgade et al., 2015). Survival status was ascertained through clinic visits or telephone follow-up. Follow-up was completed on 1 June 2025. Data collected during follow-up included the presence of locoregional or distant lymph node recurrence, distant metastasis, the time to and site of first recurrence or metastasis, the timing of initial salvage therapy following first recurrence or metastasis, the time to subsequent disease progression after initial salvage therapy, and the patient’s survival status at the time of last follow-up.

### 2.6 Subgroup analysis on platinum-based salvage therapy

To assess the differential response to conventional salvage therapy, we performed a prespecified subgroup analysis of patients who: (i) had received (neo)adjuvant chemotherapy containing both anthracyclines and taxanes (either in combination or sequentially); (ii) subsequently developed recurrent or metastatic disease; and (iii) received first-line platinum-containing chemotherapy (including cisplatin or carboplatin) as their initial salvage regimen. This subgroup was selected to evaluate whether the rrTNBC phenotype predicts resistance to a standard-of-care salvage backbone after prior anthracycline/taxane exposure (Hu et al., 2015; Yu et al., 2020).

### 2.7 Statistical analysis

Statistical analyses were conducted using R version 4.1.0 (R Foundation for Statistical Computing, Vienna, Austria). Between-group comparisons were performed using the t-test for continuous variables and the χ^2^ test or Fisher’s exact test for categorical variables. Survival curves were generated using the Kaplan-Meier method and compared using the log-rank test. All statistical tests were two-sided, and a p-value <0.05 was considered statistically significant.

## 3 Results

### 3.1 Baseline clinicopathological characteristics and recurrence patterns

A total of 638 recurrent/metastatic TNBC patients met the eligibility criteria, including 478 (74.9%) in the rrTNBC group and 160 (25.1%) in the srTNBC group. Significant differences in baseline clinicopathological features were observed between the two groups (Table 1). Compared to srTNBC patients, rrTNBC patients had a significantly higher proportion of advanced pathological TNM stage (p < 0.001), T stage (p < 0.001), and N stage (p < 0.001). Notably, rrTNBC was associated with a striking predominance of low sTIL expression (62.97% vs. 31.25%, p < 0.001). Conversely, srTNBC patients more frequently exhibited intermediate or high sTIL expression (56.25% and 12.50%, respectively) compared to rrTNBC patients (30.75% and 6.28%, respectively).

**TABLE 1:** Univariate comparison of recurrence and metastasis characteristics between rapid relapse TNBC (RR-TNBC) and slow relapse TNBC (SR-TNBC) patients.

Variables	Total (n = 638)	RR-TNBC (n = 478)	SR-TNBC (n = 160)	P value
Age at diagnosis	​	​	​	0.216
<50 years	334 (52.35)	257 (53.77)	77 (48.12)	​
≥50 years	304 (47.65)	221 (46.23)	83 (51.88)	​
Menstrual status	​	​	​	0.931
Premenopausal	357 (55.96)	267 (55.86)	90 (56.25)	​
Postmenopausal	281 (44.04)	211 (44.14)	70 (43.75)	​
Family history	​	​	​	0.560
No	583 (91.38)	435 (91.00)	148 (92.50)	​
Yes	55 (8.62)	43 (9.00)	12 (7.50)	​
Surgical approach	​	​	​	0.991
Radical surgery	602 (94.36)	451 (94.35)	151 (94.38)	​
Breast-conserving surgery	36 (5.64)	27 (5.65)	9 (5.62)	​
Pathological pattern	​	​	​	0.188
Invasive ductal carcinoma	572 (89.66)	423 (88.49)	149 (93.12)	​
Metaplastic	32 (5.02)	28 (5.86)	4 (2.50)	​
Others	34 (5.33)	27 (5.65)	7 (4.38)	​
Postoperative pathological TNM staging	​	​	​	<0.001
0 + I	87 (13.64)	51 (10.67)	36 (22.50)	​
II	304 (47.65)	215 (44.98)	89 (55.62)	​
III	247 (38.71)	212 (44.35)	35 (21.88)	​
Postoperative pathological T staging	​	​	​	<0.001
T0/Tis + T1	199 (31.19)	121 (25.31)	78 (48.75)	​
T2	379 (59.40)	301 (62.97)	78 (48.75)	​
T3+T4	60 (9.40)	56 (11.72)	4 (2.50)	​
Postoperative pathological N staging	​	​	​	<0.001
N0	227 (35.58)	152 (31.80)	75 (46.88)	​
N1	177 (27.74)	123 (25.73)	54 (33.75)	​
N2	92 (14.42)	72 (15.06)	20 (12.50)	​
N3	142 (22.26)	131 (27.41)	11 (6.88)	​
Tumor grade	​	​	​	0.997
G1+G2	295 (46.24)	221 (46.23)	74 (46.25)	​
G3	343 (53.76)	257 (53.77)	86 (53.75)	​
Lymph-vascular invasion	​	​	​	0.261
No	259 (40.60)	188 (39.33)	71 (44.38)	​
Yes	379 (59.40)	290 (60.67)	89 (55.62)	​
sTIL expression levels	​	​	​	<0.001
Low	351 (55.02)	301 (62.97)	50 (31.25)	​
Intermediate	237 (37.15)	147 (30.75)	90 (56.25)	​
High	50 (7.84)	30 (6.28)	20 (12.50)	​
Her2 expression levels	​	​	​	0.189
IHC 0	229 (35.89)	174 (36.40)	55 (34.38)	​
IHC 1+	288 (45.14)	207 (43.31)	81 (50.62)	​
IHC 2+/Fish-	121 (18.97)	97 (20.29)	24 (15.00)	​
Ki67	​	​	​	0.444
Ki67 ≤ 20	66 (10.34)	52 (10.88)	14 (8.75)	​
Ki67 > 20	572 (89.66)	426 (89.12)	146 (91.25)	​
P53	​	​	​	0.933
Negative	257 (40.28)	193 (40.38)	64 (40.00)	​
Positive	381 (59.72)	285 (59.62)	96 (60.00)	​
CK5/6	​	​	​	0.566
Negative	145 (22.73)	106 (22.18)	39 (24.38)	​
Positive	493 (77.27)	372 (77.82)	121 (75.62)	​
EGFR	​	​	​	0.374
Negative	128 (20.06)	92 (19.25)	36 (22.50)	​
Positive	510 (79.94)	386 (80.75)	124 (77.50)	​
Radiation therapy status	​	​	​	0.822
No	414 (64.89)	309 (64.64)	105 (65.62)	​
Yes	224 (35.11)	169 (35.36)	55 (34.38)	​
Chemotherapy drugs used	​	​	​	0.235
Anthracyclines	20 (3.13)	17 (3.56)	3 (1.88)	​
Taxanes	37 (5.80)	29 (6.07)	8 (5.00)	​
Anthracyclines + taxanes	531 (83.23)	391 (81.80)	140 (87.50)	​
Combined with platinum	39 (6.11)	30 (6.28)	9 (5.62)	​
None	11 (1.72)	11 (2.30)	0 (0.00)	​
Visceral metastases as the first recurrence or metastasis event	​	​	​	<0.001
No	244 (38.24)	120 (25.10)	124 (77.50)	​
Yes	394 (61.76)	358 (74.90)	36 (22.50)	​
Bone metastasis as the first recurrence or metastasis event	​	​	​	0.357
No	477 (74.76)	353 (73.85)	124 (77.50)	​
Yes	161 (25.24)	125 (26.15)	36 (22.50)	​
Brain metastasis as the first recurrence or metastasis event	​	​	​	<0.001
No	585 (91.69)	426 (89.12)	159 (99.38)	​
Yes	53 (8.31)	52 (10.88)	1 (0.62)	​
Chest wall or regional lymph nodes relapse as the first recurrence or metastasis event	​	​	​	<0.001
No	197 (30.88)	181 (37.87)	16 (10.00)	​
Yes	441 (69.12)	297 (62.13)	144 (90.00)	​
The patterns of first recurrence or metastasis	​	​	​	<0.001
Chest wall or regional lymph node recurrence prior to distant metastasis	148 (23.20)	33 (6.90)	115 (71.88)	​
Direct distant metastasis	490 (76.80)	445 (93.10)	45 (28.12)	​

Family history, HBOC-related cancer history.

CK5/6: cytokeratin 5/6; HER2: human epidermal growth factor receptor 2.

FISH: fluorescence *in situ* hybridization; EGFR: epidermal growth factor receptor.

Marked differences were also found in the patterns of first recurrence or metastasis event (Table 1). rrTNBC patients were significantly more likely to present with visceral metastases as the first recurrence or metastasis event (74.90% vs. 22.50%, p < 0.001) and brain metastases as the first recurrence or metastasis event (10.88% vs. 0.62%, p < 0.001). In contrast, srTNBC patients had a substantially higher incidence of first relapse in the chest wall or regional lymph nodes as the first recurrence or metastasis event (90.00% vs. 62.13%, p < 0.001). Consequently, the rrTNBC group exhibited a significantly higher rate of direct distant metastasis as the patterns of first recurrence or metastasis compared to the srTNBC group (93.10% vs. 28.12%, p < 0.001). No significant differences were observed between groups regarding bone metastasis as the first recurrence or metastasis event (p = 0.357), tumor grade (p = 0.997), or HER2 expression levels (p = 0.189).

### 3.2 Survival outcomes in the entire cohort

**FIGURE 1:**
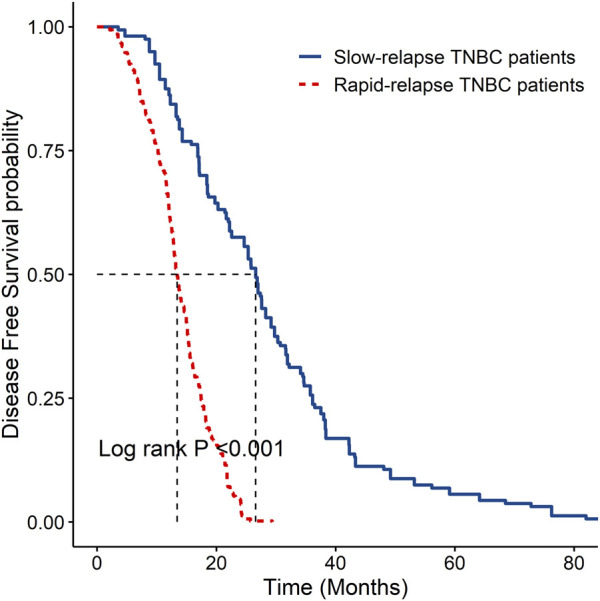
Kaplan-Meier curves of disease-free survival (DFS) in rrTNBC versus srTNBC patients.

**FIGURE 2:**
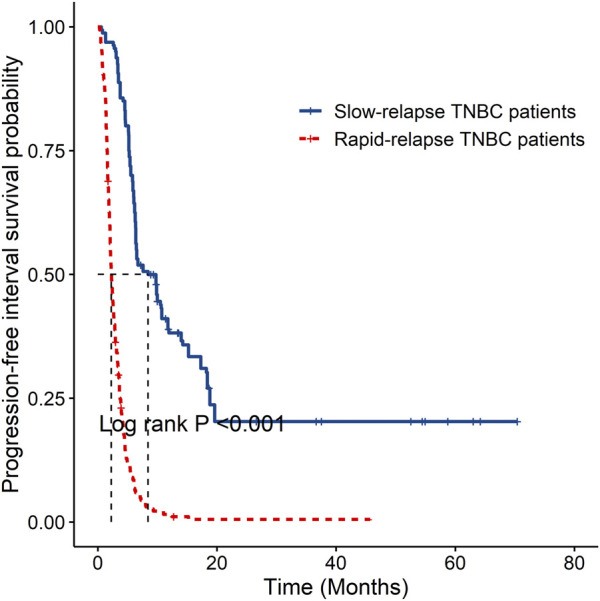
Kaplan-Meier curves of progression-free interval (PFI) after first-line salvage therapy for first recurrence or metastasis in rrTNBC versus srTNBC patients.

**FIGURE 3:**
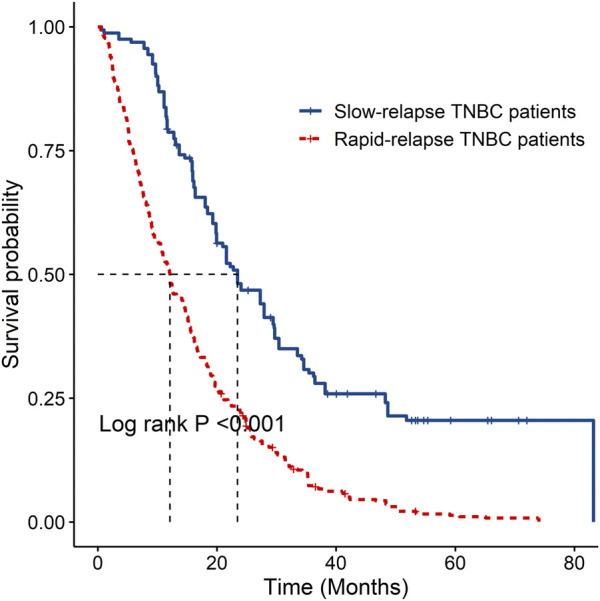
Kaplan-Meier curves of post-recurrence survival (PRS) in rrTNBC versus srTNBC patients.

**FIGURE 4:**
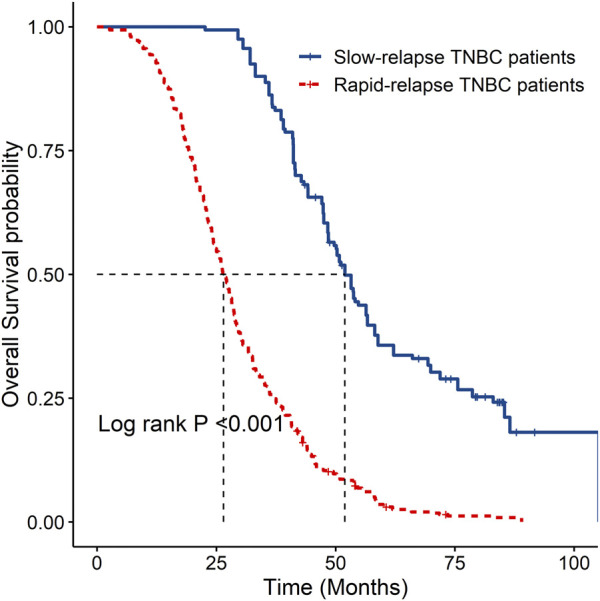
Kaplan-Meier curves of overall survival (OS) in rrTNBC versus srTNBC patients.

rrTNBC was associated with a significantly poorer prognosis across all survival metrics (Table 2). Compared to srTNBC patients, rrTNBC patients had a significantly shorter median DFS (13.4 months [95% CI, 13.0–14.0] vs. 26.6 months [95% CI, 24.7–28.3], log-rank p < 0.001, Figure 1), median PFI after first-line salvage therapy (2.27 months [95% CI, 2.13–2.50] vs. 8.43 months [95% CI, 6.37–10.7], p < 0.001, Figure 2), median PRS (12.1 months [95% CI, 10.9–13.8] vs. 23.5 months [95% CI, 19.9–27.9], p < 0.001, Figure 3), and median OS (26.5 months [95% CI, 25.5–27.9] vs. 52.0 months [95% CI, 48.5–56.7], p < 0.001, Figure 4). The Kaplan-Meier curves demonstrated early and sustained separation between the two groups for all endpoints, indicating that the poor prognosis of rrTNBC is not merely a function of earlier recurrence but reflects a fundamentally more aggressive disease biology.

**TABLE 2:** Prognostic outcomes of patients with postoperative recurrence or metastasis: comparison between rrTNBC and srTNBC in the entire cohort.

Variables	RR-TNBC (n = 478)	SR-TNBC (n = 160)	Total (n = 638)	P value
Disease-free survival (Months)	13.4 (13.0, 14.0)	26.6 (24.7, 28.3)	15.0 (14.1, 15.4)	<0.001
Progression-free interval (PFI) after first-line salvage therapy for first recurrence or metastasis (Months)	2.27 (2.13, 2.50)	8.43 (6.37, 10.7)	3.1 (2.87, 3.43)	<0.001
Post-recurrence survival (PRS) (Months)	12.1 (10.9, 13.8)	23.5 (19.9, 27.9)	14.7 (13, 15.9)	<0.001
Overall survival (Months)	26.5 (25.5, 27.9)	52.0 (48.5, 56.7)	31.7 (29.5, 33.2)	<0.001

### 3.3 Subgroup analysis: response to platinum-based salvage therapy

**FIGURE 5:**
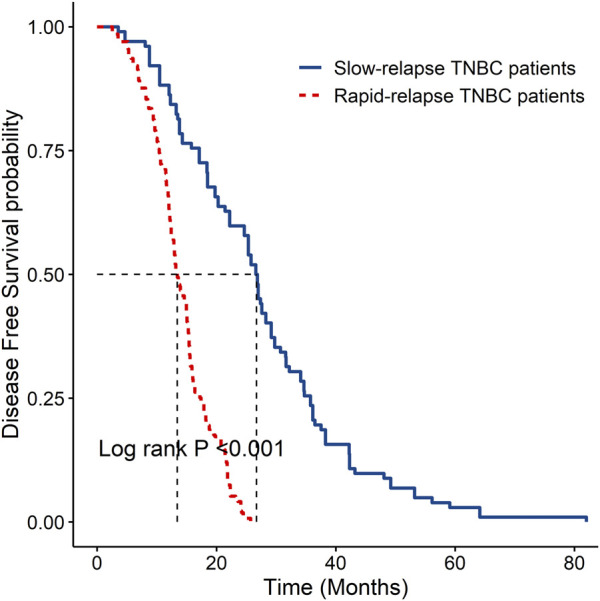
Kaplan-Meier curves of disease-free survival (DFS) in rrTNBC versus srTNBC patients who received first-line platinum-based salvage therapy after prior anthracycline/taxane treatment.

**FIGURE 6:**
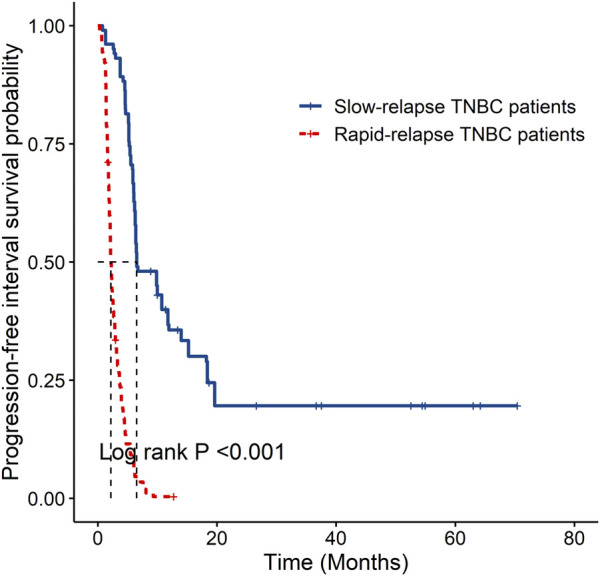
Kaplan-Meier curves of progression-free interval (PFI) after first-line salvage therapy for first recurrence or metastasis in rrTNBC versus srTNBC patients who received first-line platinum-based salvage therapy after prior anthracycline/taxane treatment.

**FIGURE 7:**
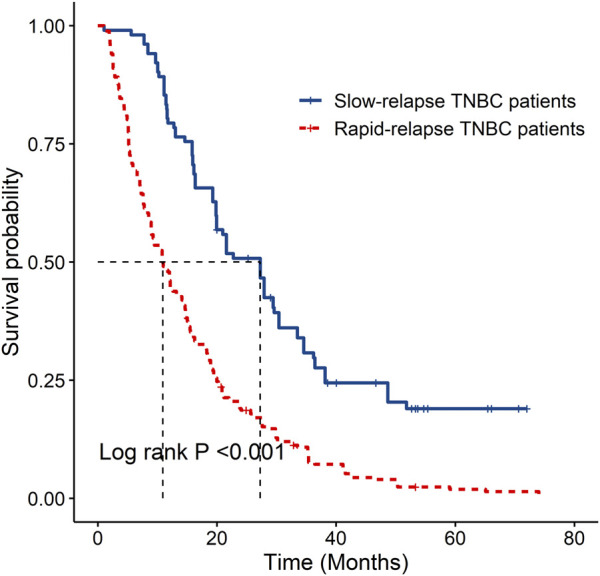
Kaplan-Meier curves of post-recurrence survival (PRS) in rrTNBC versus srTNBC patients who received first-line platinum-based salvage therapy after prior anthracycline/taxane treatment.

**FIGURE 8:**
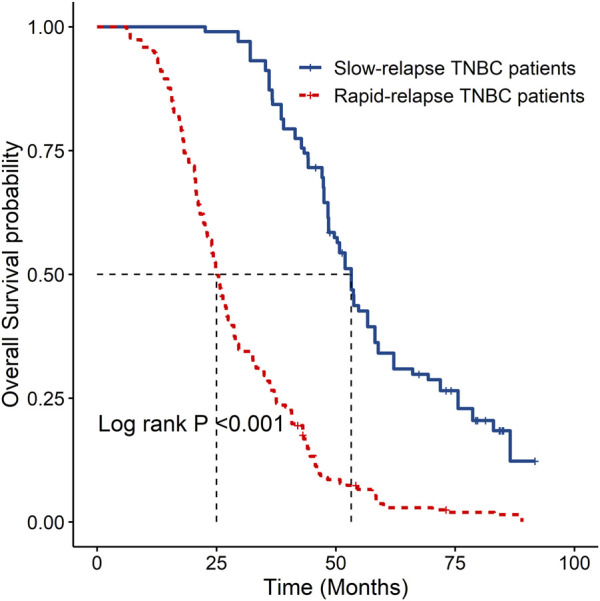
Kaplan-Meier curves of overall survival (OS) in rrTNBC versus srTNBC patients who received first-line platinum-based salvage therapy after prior anthracycline/taxane treatment.

To determine whether the poor outcomes in rrTNBC are refractory to standard salvage strategies, we analyzed a subgroup of 369 patients who had progressed on (neo)adjuvant anthracycline/taxane therapy and subsequently received first-line platinum-based chemotherapy (Table 3). Within this subgroup, rrTNBC patients (n = 267) continued to show significantly worse outcomes compared to srTNBC patients (n = 102) across all endpoints: median DFS (13.4 months [95% CI, 12.9–15.0] vs. 26.7 months [95% CI, 24.7–29.2], p < 0.001, Figure 5), PFI on platinum-based salvage therapy (2.20 months [95% CI, 2.10–2.43] vs. 6.53 months [95% CI, 6.27–11.77], p < 0.001, Figure 6), PRS (10.9 months [95% CI, 9.27–13.2] vs. 27.3 months [95% CI, 19.93–30.4], p < 0.001, Figure 7), and OS (25.0 months [95% CI, 24.1–26.9] vs. 53.2 months [95% CI, 48.5–58.2], p < 0.001, Figure 8). The median PFI of only 2.20 months in rrTNBC patients receiving platinum-based therapy underscores the profound chemoresistance of this aggressive subtype.

**TABLE 3:** Prognostic outcomes of patients who developed recurrence or metastasis after (neo)adjuvant anthracycline/taxane-based therapy and received first-line platinum-based salvage therapy: comparison between rrTNBC and srTNBC.

Variables	RR-TNBC (n = 267)	SR-TNBC (n = 102)	Total (n = 369)	P value
Disease-free survival (Months)	13.4 (12.9,15.0)	26.7 (24.7, 29.2)	15.1 (14.1,15.6)	<0.001
Progression-free interval (PFI) after first-line salvage therapy for first recurrence or metastasis (Months)	2.20 (2.10,2.43)	6.53 (6.27,11.77)	3.03 (2.67,3.63)	<0.001
Post-recurrence survival (PRS) (Months)	10.9 (9.27,13.2)	27.3 (19.93,30.4)	14.6 (12.2,16.1)	<0.001
Overall survival (Months)	25.0 (24.1,26.9)	53.2 (48.5,58.2)	32.6 (28.7,36.2)	<0.001

## 4 Discussion

To our knowledge, this study provides the most comprehensive clinical characterization of the rrTNBC phenotype to date, leveraging a large, real-world cohort of 638 patients with recurrent or metastatic disease. Our findings demonstrate that rrTNBC is not merely an earlier recurrence of typical TNBC but rather a clinically distinct phenotype, defined by a unique constellation of clinicopathological features, recurrence patterns, and-most critically-markedly poorer outcomes with conventional platinum-based salvage therapy. This phenotype-treatment interaction, rather than demonstrating absolute refractoriness to all cytotoxic agents, suggests a differential sensitivity profile that reflects underlying biological heterogeneity between rrTNBC and srTNBC.

First, our data reveal that rrTNBC is characterized by a more aggressive baseline profile, including significantly higher T, N, and TNM stages, as well as a marked predominance of low sTIL expression. The association between low sTILs and rapid relapse aligns with the emerging paradigm that an immunosuppressive tumor microenvironment (TME) is a key driver of poor outcomes in TNBC (Loi et al., 2019; Gao et al., 2020). Low sTIL levels indicate a paucity of effector T cells, which may permit the early emergence and unchecked expansion of highly invasive tumor clones (Park et al., 2019). Recent studies have demonstrated that sTIL-rich TNBCs are associated with higher rates of pathological complete response to neoadjuvant chemotherapy and improved long-term survival, whereas sTIL-poor tumors exhibit a “cold” immune phenotype that is permissive to metastatic progression (T de Jong et al., 2022). Our findings extend these observations by demonstrating that low sTIL expression is specifically associated with the rapid relapse phenotype, suggesting that the baseline immune landscape may be a critical determinant of the tempo of disease progression. This is consistent with the genomic analysis by Zhang et al., who found that rapid relapse was associated with significantly lower expression of immune-related gene signatures (Zhang et al., 2021). Collectively, these data support the hypothesis that rrTNBC may arise from clones that have evolved mechanisms to evade immune surveillance, thereby facilitating early dissemination (Binnew et al., 2018).

Second, we identified a distinct metastatic organotropism in rrTNBC, characterized by a striking predilection for direct visceral (lung/liver) and brain metastases, with a correspondingly lower incidence of locoregional recurrence as the first event. Over 93% of rrTNBC patients presented with direct distant metastasis at first recurrence, compared to only 28% of srTNBC patients. This pattern is clinically ominous, as visceral and brain metastases are associated with substantially worse prognoses and limited treatment options compared to locoregional or bone-only recurrences (Lin et al., 2012; Bu and Bo, 2026). The biological basis for this organotropism is poorly understood but may involve the early acquisition of specific genetic or epigenetic alterations that confer the ability to colonize distant organs. For example, activation of the PI3K/AKT pathway and dysregulation of the blood-brain barrier have been implicated in brain metastasis (Witzel et al., 2016). Furthermore, the absence of a “warning” locoregional relapse in rrTNBC suggests that the metastatic cascade-including intravasation, circulation, extravasation, and colonization-occurs remarkably early in the disease course, potentially even prior to initial diagnosis (Kavan et al., 2022). This has profound clinical implications, as it suggests that locoregional therapies alone (surgery and radiation) are unlikely to be curative for patients with occult micrometastases at presentation, a scenario that appears to be the rule rather than the exception in rrTNBC.

Third, and perhaps most importantly from a clinical management perspective, our subgroup analysis demonstrated that rrTNBC is characterized by profound resistance to platinum-based salvage therapy. Among patients who had progressed on standard (neo)adjuvant anthracycline/taxane regimens, those with rrTNBC derived minimal benefit from first-line platinum-containing chemotherapy, with a median PFI of only 2.20 months and a median PRS of only 10.9 months. This contrasts sharply with the srTNBC patients, who had a median PFI of 6.53 months and a median PRS of 27.3 months on the same regimen. These findings indicate that the aggressive clones driving rrTNBC are either inherently cross-resistant to multiple classes of conventional cytotoxic agents (anthracyclines, taxanes, and platinum compounds) or rapidly acquire such resistance through evolutionary mechanisms (Cortazar et al., 2014). This is consistent with the concept of “vertical” or “horizontal” clonal selection, where pre-existing or *de novo* resistant clones expand under therapeutic pressure (Burrell et al., 2013). The clinical implication is stark: conventional salvage chemotherapy is largely ineffective for rrTNBC patients, and continued reliance on such approaches is unlikely to improve outcomes.

Taken together, our findings support a model of rapid clonal evolution in rrTNBC (Figure 3). We hypothesize that at diagnosis, TNBC tumors already harbor a high degree of intratumoral heterogeneity, with pre-existing subclones possessing aggressive traits such as immune evasion (low sTILs), metastatic organotropism (visceral/brain tropism), and multidrug resistance (Barj et al., 2026). Standard (neo)adjuvant chemotherapy effectively eliminates chemosensitive clones but exerts strong selective pressure that allows these pre-existing aggressive clones to expand and dominate the post-treatment tumor landscape, leading to rapid distant relapse. The failure of first-line platinum-based therapy further supports the concept that the dominant clones in rrTNBC are inherently resistant to conventional DNA-damaging agents, potentially due to upregulated DNA repair pathways (e.g., PARP, FANCI) or alterations in drug efflux transporters (Huang et al., 2025).

These findings have urgent and actionable implications for clinical practice and trial design. First, risk stratification incorporating T stage, N stage, and sTIL expression at diagnosis could identify patients at high risk for rapid relapse, who should be prioritized for closer surveillance and more intensive initial therapy. Second, novel therapeutic strategies are desperately needed for rrTNBC patients. Given the resistance to platinum agents, future trials should evaluate more innovative approaches as first-line salvage therapy, rather than reserving them for later lines. Promising candidates include:

Antibody-Drug Conjugates (ADCs): Sacituzumab govitecan, a Trop-2-directed ADC, has shown remarkable activity in pretreated metastatic TNBC, with a median PFS(progression free survival) of 5.6 months and OS of 12.1 months in the ASCENT trial (Bardia et al., 2021). Similarly, trastuzumab deruxtecan has demonstrated efficacy in HER2-low breast cancer, a category that includes a substantial proportion of TNBC patients (Modi et al., 2022). Given the poor outcomes with platinum, these agents should be evaluated as first-line therapy for rrTNBC.

Immunotherapy Combinations: Although rrTNBC is characterized by low sTILs (a “cold” TME), combination strategies that convert cold tumors into hot tumors may be effective. The KEYNOTE-355 trial demonstrated a PFS benefit for pembrolizumab plus chemotherapy in PD-L1 positive (CPS≥10) metastatic TNBC (Cortes et al., 2020). Ongoing trials of dual immunotherapy (e.g., anti-PD-1 plus anti-CTLA-4) or combinations with ADCs are warranted in rrTNBC.

PARP Inhibitors: For the subset of rrTNBC patients with germline BRCA1/2 mutations (approximately 15%–20% of TNBC), PARP inhibitors such as olaparib and talazoparib have shown efficacy (Robson et al., 2017; Litton et al., 2018). The OlympiAD trial demonstrated a PFS benefit for olaparib over chemotherapy of physician’s choice in gBRCA-mutated HER2-negative metastatic breast cancer (Robson et al., 2017). Genotyping should be performed routinely in rrTNBC patients.

An important methodological consideration in interpreting our platinum-subgroup analysis is the heterogeneity of the denominator population. The entire cohort of 638 recurrent/metastatic TNBC patients included not only those who had received prior anthracycline/taxane-based (neo)adjuvant therapy followed by first-line platinum-based salvage (n = 369, the platinum-subgroup), but also patients with diverse prior exposure patterns and salvage regimens. The fact that rrTNBC versus srTNBC differences in median DFS, PFI, PRS, and OS remained highly significant (all p < 0.001) in both the overall cohort and the platinum-subgroup indicates that the prognostic impact of the rrTNBC phenotype is robust and independent of treatment line or regimen selection. However, the similar magnitude of survival separation between the overall and platinum-subgroup analyses does not imply that platinum therapy is ineffective; rather, it highlights that rrTNBC patients, irrespective of salvage regimen, exhibit an inherently more aggressive clinical course. Importantly, these findings do not negate the role of platinum agents as a guideline-recommended salvage option for metastatic TNBC (Hu et al., 2015; Sipos et al., 2021; Wang et al., 2022), but they strongly suggest that rrTNBC patients may derive disproportionately limited benefit and should therefore be prioritised for novel therapeutic strategies, including antibody–drug conjugates (ADCs) and immuno-oncology combinations, in future prospective trials. This interpretation is consistent with the clonal evolution model we propose, wherein pre-existing aggressive subclones drive early dissemination and multidrug tolerance, rather than acquired resistance to a single agent class.

## 5 Study limitations

Several limitations of this study warrant acknowledgment. First, the retrospective, single-center design may introduce selection bias, and our findings may not be generalizable to other populations or healthcare settings. Second, the definition of rrTNBC based on a 24-month cut-off, while clinically relevant and consistent with prior literature (Asad et al., 2021; Zhang et al., 2021; Obeng-Gyasi et al., 2021), is arbitrary; the biology likely exists on a continuum. Third, detailed genomic or transcriptomic data were not available for most patients, limiting our ability to directly link clinical phenotypes to specific evolutionary mechanisms. Fourth, although we analysed the platinum-based salvage subgroup, the regimens after recurrence were otherwise heterogeneous, and we lacked systematic collection of treatment-related adverse events and dose modifications. Fifth, and most importantly, we did not collect data on patient-reported outcomes (PROs) or quality of life, which are increasingly recognised as co-primary endpoints in metastatic disease trials given the trade-off between survival gains and substantial treatment-related toxicities (Pe et al., 2023). Sixth, the observational nature of our study precludes any causal inference; the significant survival separation observed on Kaplan-Meier analysis demonstrates association, not causation. Finally, the follow-up period, while sufficient to capture the majority of rapid relapse events (given that >80% of TNBC recurrences occur within 3 years, may be inadequate to fully characterise the long-term outcomes of srTNBC patients, some of whom may experience late relapse beyond 5 years (Pedersen et al., 2022). Future prospective, multicentre studies with comprehensive genomic profiling, centralised pathological review, standardised treatment protocols, integrated PRO endpoints, and extended follow-up are urgently needed to validate our findings and elucidate the molecular drivers of rapid relapse.

## 6 Conclusion

In conclusion, rrTNBC is a highly aggressive and clinically distinct subtype of TNBC characterized by advanced stage, low sTIL expression, a predilection for direct visceral and brain metastasis, and profound resistance to conventional platinum-based salvage therapy. These findings provide a clinical framework for understanding the rapid clonal evolution that drives this lethal phenotype. We propose that rrTNBC patients be considered a distinct therapeutic subset for whom novel strategies-including ADCs, immunotherapy combinations, and PARP inhibitors-should be evaluated as first-line salvage therapy rather than conventional chemotherapy. Such a paradigm shift has the potential to meaningfully improve outcomes for patients with this devastating disease.

## Data Availability

The original contributions presented in the study are included in the article/supplementary material, further inquiries can be directed to the corresponding authors.

## References

[B1] Aguilar-Mahecha A. Alirezaie N. Lafleur J. Bareke E. Przybytkowski E. Lan C. (2023). The mutational spectrum of Pre- and post-neoadjuvant chemotherapy triple-negative breast cancers. Genes (Basel) 15 (1), 27. 10.3390/genes15010027 38254917 PMC10815241

[B2] Asad S. Barcenas C. H. Bleicher R. J. Cohen A. L. Javid S. H. Levine E. G. (2021). Sociodemographic factors associated with rapid relapse in triple-negative breast cancer: a multi-institution study. J. Natl. Compr. Canc Netw. 19 (7), 797–804. 10.6004/jnccn.2020.7659 33691275 PMC8564718

[B3] Bardia A. Hurvitz S. A. Tolaney S. M. Loirat D. Punie K. Oliveira M. (2021). Sacituzumab govitecan in metastatic triple-negative breast cancer. N. Engl. J. Med. 384 (16), 1529–1541. 10.1056/NEJMoa2028485 33882206

[B4] Barjij I. Lamsyah O. Kdadri S. Lkhoyaali S. Najem S. Naciri S. (2026). Spatial and temporal intratumoral heterogeneity in breast cancer: a systematic and conceptual review of single-cell and spatial omics studies. BMC Cancer 26 (1), 639. 10.1186/s12885-026-15928-0 41942920 PMC13188440

[B5] Bauer K. R. Brown M. Cress R. D. Parise C. A. Caggiano V. (2007). Descriptive analysis of estrogen receptor (ER)-negative, progesterone receptor (PR)-negative, and HER2-negative invasive breast cancer, the so-called triple-negative phenotype: a population-based study from the California cancer registry. Cancer 109 (9), 1721–1728. 10.1002/cncr.22618 17387718

[B6] Binnewies M. Roberts E. W. Kersten K. Chan V. Fearon D. F. Merad M. (2018). Understanding the tumor immune microenvironment (TIME) for effective therapy. Nat. Med. 24 (5), 541–550. 10.1038/s41591-018-0014-x 29686425 PMC5998822

[B7] Bray F. Laversanne M. Sung H. Ferlay J. Siegel R. L. Soerjomataram I. (2024). Global cancer statistics 2022: GLOBOCAN estimates of incidence and mortality worldwide for 36 cancers in 185 countries. CA Cancer J. Clin. 74 (3), 229–263. 10.3322/caac.21834 38572751

[B8] Bu R. Bo L. (2026). Orchestrating organotropism: miRNA-driven mechanisms of site-specific metastasis in triple-negative breast cancer. Oncol. Lett. 31 (5), 151. 10.3892/ol.2026.15504 41822558 PMC12977313

[B9] Burrell R. A. McGranahan N. Bartek J. Swanton C. (2013). The causes and consequences of genetic heterogeneity in cancer evolution. Nature 501 (7467), 338–345. 10.1038/nature12625 24048066

[B10] Cortazar P. Zhang L. J. Untch M. Mehta K. Costantino J. P. Wolmark N. (2014). Pathological complete response and long-term clinical benefit in breast cancer: the CTNeoBC pooled analysis. Lancet 384 (9938), 164–172. 10.1016/S0140-6736(13)62422-8 24529560

[B11] Cortes J. Cescon D. W. Rugo H. S. Nowecki Z. Im S. A. Yusof M. M. (2020). Pembrolizumab plus chemotherapy versus placebo plus chemotherapy for previously untreated locally recurrent inoperable or metastatic triple-negative breast cancer (KEYNOTE-355): a randomised, placebo-controlled, double-blind, phase 3 clinical trial. Lancet 396 (10265), 1817–1828. 10.1016/S0140-6736(20)32531-9 33278935

[B12] Dent R. Trudeau M. Pritchard K. I. Hanna W. M. Kahn H. K. Sawka C. A. (2007). Triple-negative breast cancer: clinical features and patterns of recurrence. Clin. Cancer Res. 13 (15 Pt 1), 4429–4434. 10.1158/1078-0432.CCR-06-3045 17671126

[B13] Dent R. Hanna W. M. Trudeau M. Rawlinson E. Sun P. Narod S. A. (2009). Pattern of metastatic spread in triple-negative breast cancer. Breast Cancer Res. Treat. 115 (2), 423–428. 10.1007/s10549-008-0086-2 18543098

[B14] Foulkes W. D. Smith I. E. Reis-Filho J. S. (2010). Triple-negative breast cancer. N. Engl. J. Med. 363 (20), 1938–1948. 10.1056/NEJMra1001389 21067385

[B15] Gao G. X. Wang Z. H. Qu X. Zhang Z. T. (2020). Prognostic value of tumor-infiltrating lymphocytes in patients with triple-negative breast cancer: a systematic review and meta-analysis. BMC Cancer 20 (1), 179. 10.1186/s12885-020-6668-z 32131780 PMC7057662

[B16] Gourgou-Bourgade S. Cameron D. Poortmans P. Asselain B. Azria D. Cardoso F. (2015). Guidelines for time-to-event end point definitions in breast cancer trials: results of the DATECAN initiative (definition for the assessment of time-to-event endpoints in CANcer trials). Ann. Oncol. 26 (5), 873–879. 10.1093/annonc/mdv106 25725046

[B17] Harbeck N. Gnant M. (2017). Breast cancer. Lancet. 389 (10074), 1134–1150. 10.1016/S0140-6736(16)31891-8 27865536

[B18] Hu X. C. Zhang J. Xu B. H. Cai L. Ragaz J. Wang Z. H. (2015). Cisplatin plus gemcitabine versus paclitaxel plus gemcitabine as first-line therapy for metastatic triple-negative breast cancer (CBCSG006): a randomised, open-label, multicentre, phase 3 trial. Lancet Oncol. 16 (4), 436–446. 10.1016/S1470-2045(15)70064-1 25795409

[B19] HuangS. S. QiuY. WuL. Y. XieY. HeZ. T. LiY. Q. (2025). ZUP1 promotes DNA repair and immune evasion to drive olaparib resistance in triple-negative breast cancer. J. Adv. Res. 16 (25), S2090. 10.1016/j.jare.2025.11.038 41253271

[B20] Hudis C. A. Barlow W. E. Costantino J. P. Gray R. J. Pritchard K. I. Chapman J. A. W. (2007). Proposal for standardized definitions for efficacy end points in adjuvant breast cancer trials: the STEEP system. J. Clin. Oncol. 25 (15), 2127–2132. 10.1200/JCO.2006.10.3523 17513820

[B21] Kavan S. Kruse T. A. Vogsen M. Hildebrandt M. G. Thomassen M. (2022). Heterogeneity and tumor evolution reflected in liquid biopsy in metastatic breast cancer patients: a review. Cancer Metastasis Rev. 41 (2), 433–446. 10.1007/s10555-022-10023-9 35286542

[B22] Liedtke C. Mazouni C. Hess K. R. André F. Tordai A. Mejia J. A. (2008). Response to neoadjuvant therapy and long-term survival in patients with triple-negative breast cancer. J. Clin. Oncol. 26 (8), 1275–1281. 10.1200/JCO.2007.14.4147 18250347

[B23] Lin N. U. Vanderplas A. Hughes M. E. Theriault R. L. Edge S. B. Wong Y. N. (2012). Clinicopathologic features, patterns of recurrence, and survival among women with triple-negative breast cancer in the national comprehensive cancer network. Cancer 118 (22), 5463–5472. 10.1002/cncr.27581 22544643 PMC3611659

[B24] Litton J. K. Rugo H. S. Ettl J. Hurvitz S. A. Gonçalves A. Lee K. H. (2018). Talazoparib in patients with advanced breast cancer and a germline BRCA mutation. N. Engl. J. Med. 379 (8), 753–763. 10.1056/NEJMoa1802905 30110579 PMC10600918

[B25] Loi S. Drubay D. Adams S. Pruneri G. Francis P. A. Lacroix-Triki M. (2019). Tumor-infiltrating lymphocytes and prognosis: a pooled individual patient analysis of early-stage triple-negative breast cancers. J. Clin. Oncol. 37 (7), 559–569. 10.1200/JCO.18.01010 30650045 PMC7010425

[B26] McGranahan N. Swanton C. (2017). Clonal heterogeneity and tumor evolution: past, present, and the future. Cell 168 (4), 613–628. 10.1016/j.cell.2017.01.018 28187284

[B27] Modi S. Jacot W. Yamashita T. Sohn J. Vidal M. Tokunaga E. (2022). Trastuzumab deruxtecan in previously treated HER2-Low advanced breast cancer. N. Engl. J. Med. 387 (1), 9–20. 10.1056/NEJMoa2203690 35665782 PMC10561652

[B28] Obeng-Gyasi S. Asad S. Fisher J. L. Rahurkar S. Stover D. G. (2021). Socioeconomic and surgical disparities are associated with rapid relapse in patients with triple-negative breast cancer. Ann. Surg. Oncol. 28 (11), 6500–6509. 10.1245/s10434-021-09688-3 33586064 PMC9143975

[B29] Park J. H. Jonas S. F. Bataillon G. Criscitiello C. Salgado R. Loi S. (2019). Prognostic value of tumor-infiltrating lymphocytes in patients with early-stage triple-negative breast cancers (TNBC) who did not receive adjuvant chemotherapy. Ann. Oncol. 30 (12), 1941–1949. 10.1093/annonc/mdz395 31566659

[B30] Pe M. Alanya A. Falk R. S. Amdal C. D. Bjordal K. Chang J. (2023). Setting international standards in analyzing patient-reported outcomes and quality of life endpoints in cancer clinical trials-innovative medicines initiative (SISAQOL-IMI): stakeholder views, objectives, and procedures. Lancet Oncol. 24 (6), e270–e283. 10.1016/S1470-2045(23)00157-2 37269858

[B31] Pedersen R. N. Esen B. Ö. Mellemkjær L. Christiansen P. Ejlertsen B. Lash T. L. (2022). The incidence of breast cancer recurrence 10-32 years after primary diagnosis. J. Natl. Cancer Inst. 114 (3), 391–399. 10.1093/jnci/djab202 34747484 PMC8902439

[B32] Robson M. Im S. A. Senkus E. Xu B. H. Domchek S. M. Masuda N. (2017). Olaparib for metastatic breast cancer in patients with a germline BRCA mutation. N. Engl. J. Med. 377 (6), 523–533. 10.1056/NEJMoa1706450 28578601

[B33] Salgado R. Denkert C. Demaria S. Sirtaine N. Klauschen F. Pruneri G. (2015). The evaluation of tumor-infiltrating lymphocytes (TILs) in breast cancer: recommendations by an international TILs working group 2014. Ann. Oncol. 26 (2), 259–271. 10.1093/annonc/mdu450 25214542 PMC6267863

[B34] Schmid P. Adams S. Rugo H. S. Schneeweiss A. Barrios C. H. Iwata H. (2018). Atezolizumab and nab-paclitaxel in advanced triple-negative breast cancer. N. Engl. J. Med. 379 (22), 2108–2121. 10.1056/NEJMoa1809615 30345906

[B35] Sipos O. Tovey H. Quist J. Haider S. Nowinski S. Gazinska P. (2021). Assessment of structural chromosomal instability phenotypes as biomarkers of carboplatin response in triple negative breast cancer: the TNT trial. Ann. Oncol. 32 (1), 58–65. 10.1016/j.annonc.2020.10.475 33098992 PMC7784666

[B36] T de Jong V. M. Wang Y. W. Ter Hoeve N. D. Opdam M. Stathonikos N. Jóźwiak K. (2022). Prognostic value of stromal tumor-infiltrating lymphocytes in young, node-negative, triple-negative breast cancer patients who did not receive (neo)Adjuvant systemic therapy. J. Clin. Oncol. 40 (21), 2361–2374. 10.1200/JCO.21.01536 35353548 PMC9287283

[B37] Wang B. Y. Sun T. Zhao Y. N. Wang S. S. Zhang J. Wang Z. H. (2022). A randomized phase 3 trial of gemcitabine or Nab-paclitaxel combined with cisPlatin as first-line treatment in patients with metastatic triple-negative breast cancer. Nat. Commun. 13 (1), 4025. 10.1038/s41467-022-31704-7 35821019 PMC9276725

[B38] Witzel I. Oliveira-Ferrer L. Pantel K. Müller V. Wikman H. (2016). Breast cancer brain metastases: biology and new clinical perspectives. Breast Cancer Res. 18 (1), 8. 10.1186/s13058-015-0665-1 26781299 PMC4717619

[B39] Wolff A. C. Hammond M. E. H. Allison K. H. Harvey B. E. Mangu P. B. Bartlett J. M. S. (2018). Human epidermal growth factor receptor 2 testing in breast cancer: American society of clinical oncology/college of American pathologists clinical practice guideline focused update. J. Clin. Oncol. 36 (20), 2105–2122. 10.1200/JCO.2018.77.8738 29846122

[B40] Yin W. J. Lu J. S. Di G. H. Lin Y. P. Zhou L. H. Liu G. Y. (2009). Clinicopathological features of the triple-negative tumors in Chinese breast cancer patients. Breast Cancer Res. Treat. 115 (2), 325–333. 10.1007/s10549-008-0096-0 18563552

[B41] Yu K. D. Ye F. G. He M. Fan L. Ma D. Mo M. (2020). Effect of adjuvant paclitaxel and carboplatin on survival in women with triple-negative breast cancer: a phase 3 randomized clinical trial. JAMA Oncol. 6 (9), 1390–1396. 10.1001/jamaoncol.2020.2965 32789480 PMC7426881

[B42] Zhang Y. Q. Asad S. Weber Z. Tallman D. Nock W. Wyse M. (2021). Genomic features of rapid versus late relapse in triple negative breast cancer. BMC Cancer 21 (1), 568. 10.1186/s12885-021-08320-7 34006255 PMC8130400

